# Ureidoglycolate hydrolase, amidohydrolase, lyase: how errors in biological databases are incorporated in scientific papers and vice versa

**DOI:** 10.1093/database/bat071

**Published:** 2013-10-08

**Authors:** Riccardo Percudani, Davide Carnevali, Vincenzo Puggioni

**Affiliations:** Department of Life Sciences, Laboratory of Biochemistry, Molecular Biology and Bioinformatics, University of Parma, Italy

## Abstract

An opaque biochemical definition, an insufficient functional characterization, an interpolated database description, and a beautiful 3D structure with a wrong reaction. All these are elements of an exemplar case of misannotation in biological databases and confusion in the scientific literature concerning genes and enzymes acting on ureidoglycolate, an intermediate of purine catabolism. Here we show biochemical evidence for the relocation of genes assigned to EC 3.5.3.19 (ureidoglycolate hydrolase, releasing ammonia), such as *allA* of *Escherichia coli* or *DAL3* of *Saccharomyces cerevisiae*, to EC 4.3.2.3 (ureidoglycolate lyase, releasing urea). The EC 3.5.3.19 should be more appropriately named ureidoglycolate amidohydrolase and include genes equivalent to UAH of *Arabidopsis thaliana*. The distinction between ammonia- or urea-releasing activities from ureidoglycolate is relevant for the understanding of nitrogen metabolism in various organisms and of virulence factors in certain pathogens rather than a nomenclature problem. We trace the original fault in database annotation and provide a rationale for its incorporation and persistence in the scientific literature. Notwithstanding the technological distance, yet not surprising for the constancy of human nature, error categories and mechanisms established in the study of the work of amanuensis monks still apply to the modern curation of biological databases.

## Introduction

In the middle ages, scribe monks called amanuenses contributed to the continuation of the human knowledge by copying and glossing manuscripts on paper. As documented in a rich tradition of philological studies, this work was not free of faults. Once introduced, errors could easily persist in subsequent transcriptions and eventually become set in the printed edition. The understanding of the common mechanisms of the faults of amanuenses has helped identification and correction of inaccuracies, omissions or spurious additions in written texts (1, 2).

In modern time, manual curation of databases is made to ensure transmission of biological data and knowledge. It is notorious that this ‘digital monk’s work’ is accompanied by errors (3–6). Less understood are the mechanisms by which these errors are made, persist and eventually become set in the scientific literature. Here we analyse a paradigmatic case of faulty annotation of gene and protein function in biological databases, which dates back to the beginning of ‘90s. This error has been incorporated in scientific literature and has persisted until now in spite of the opposing evidence (7, 8). Amusingly, the results of our analysis indicate that error descriptions and categories established by philological studies for the work of amanuensis monks apply well to the transmission of biological knowledge in the digital era.

Here we describe previous and original evidence to correct the wrong annotation, and discuss what can be the best practice to avoid a recurrence of the error and ensure a rapid emendation of the biological databases and scientific literature.

## Results and discussion

### The subject matter: nitrogen release from ureidoglycolate

**Figure 1.**
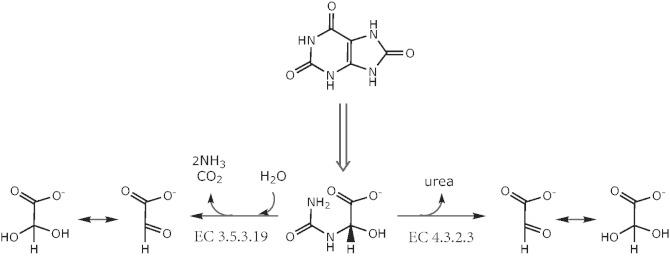
Alternative routes of nitrogen release from ureidoglycolate. The enzymatic activities involved in the reactions are indicated with the corresponding EC numbers.

In many species, ureidoglycolate (sometimes ureidoglycollate) is the last intermediate of purine breakdown. Of the uric acid double ring, the molecule conserves the two central carbon atoms and an ureido group (Figure 1); two other nitrogen atoms had been released as urea or ammonia in earlier steps of the pathway. Ureidoglycolate can be oxidized to oxalurate by the well characterized AllD protein (9), or acted on by two distinct enzymes for the release of nitrogen (Figure 1). Two moles of ammonia and one of carbon dioxide are released on the action of ureidoglycolate amidohydrolase (10) (EC 3.5.3.19), while one mole of urea is released on the action of ureidoglycolate lyase (11) (EC 4.3.2.3). In both cases, the final product is glyoxylate, which at neutral pH in aqueous solutions is almost entirely in a hydrated form (12). From a mechanistic standpoint, the reaction catalysed by EC 3.5.3.19 is unmistakeably a hydrolytic reaction caused by the attack of water to the amidic bond of ureidoglycolate, with formation of carbamate and hydroxyglycine as primary products (13). In principle, urea release from ureidoglycolate could involve a hydrolytic reaction or not, depending on whether the primary product is the hydrated or the aldehyde form of glyoxylate. In their classification of EC 4.3.2.3 as a lyase, the authors have implied that water is not involved in the reaction, though this is not biochemically proven.

Ureidoglycolate amidohydrolase was originally described in plants (10). The gene responsible for this ammonia-release activity (UAH) has been identified in *Arabidopsis thaliana* and the corresponding protein functionally characterized (14). The *A. thaliana* UAH and equivalent plant genes are correctly assigned to EC 3.5.3.19 in most biological databases.

Ureidoglycolate lyase was first described in bacteria and later reported in fungi (11, 15, 16). Genes responsible for this urea-releasing activity have been identified in *Saccharomyces cerevisiae* and *Escherichia coli* and named, respectively, *DAL3* and *allA* (sequence similarity indicates that two genes are homologous). Surprisingly, *DAL3*, *allA* and equivalent genes from bacteria and fungi are incorrectly assigned to EC 3.5.3.19 in most biological databases (see below), while in dedicated studies, the corresponding proteins are supposed to catalyse the release of ammonia instead of urea from ureidoglycolate (7, 8). How did this confusion come about? Which reaction do DAL3/ALLA proteins catalyse?

### Nickel-dependent urea-release activity of AllA (EC 4.3.2.3)

**Figure 2.**
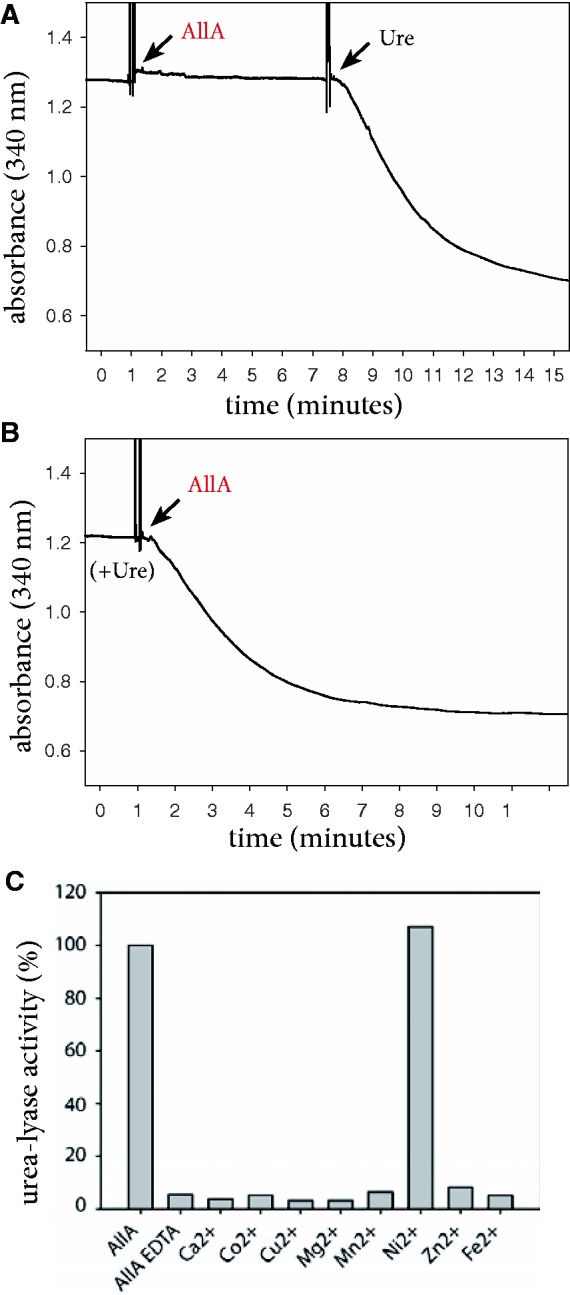
Biochemical evidence that AllA catalyses nickel-dependent urea release from ureidoglycolate. (**A**) Kinetics of ammonia release catalysed by the recombinant AllA protein before and after addition of urease; (**B**) kinetics of ammonia release with urease present in the reaction mixture; (**C**) ureidoglycolate-lyase activity of the AllA protein treated with a metal chelator (EDTA) and supplemented with various divalent ions.

Using a discontinuous assay, Werner et al. (14) reported that AllA releases urea instead of ammonia from ureidoglycolate, thus acting as an ureidoglycolate lyase. Their observations can be confirmed using a continuous assay that can unambiguously discriminate between ammonia- or urea-releasing activities (Figure 2). This experiment shows that recombinant AllA from *E. coli* is unable to release ammonia directly from ureidoglycolate; however, the formation of two moles of ammonia is observed after the addition of urease (Figure 2A). Accordingly, two moles of ammonia are produced when AllA is added to a reaction mixture containing ureidoglycolate and urease (Figure 2B). Therefore, consistent with EC 4.3.2.3, AllA catalyses release of urea from ureidoglycolate. Because of the conservation of the enzyme catalytic mechanisms (17), this property can be extended to the homologous DAL3 protein.

The AllA protein structure (8) suggests similarity with metal-dependent enzymes. Accordingly, the urea-lyase activity is abolished if the protein is incubated with metal chelators as EDTA. The activity can be only rescued by the incubation of the EDTA-treated protein with an excess of Ni^2+^ ions (Figure 2C), indicating that AllA, like urease, is a nickel-dependent enzyme. This property is at variance with other proteins of the same pathway that require zinc (18, 19) or manganese (20, 21) for the activity.

### Genes and enzymes acting on ureidoglycolate in biological databases

Notwithstanding the opposing evidence, in most biological databases DAL3/AllA genes or proteins are considered ureidoglycolate amidohydrolase. As listed in Table 1, they are typically assigned to EC 3.5.3.19, while the EC 4.3.2.3 is normally occupied by a bacterial gene (not homologous to DAL3/AllA), whose involvement in the enzymatic reaction has been called into question (23). A notable exception is the MetaCyc database (25), in which the assignments for the two enzymatic activities are in perfect agreement with our proposed assignments (Table 1).

**Table 1.** Current and proposed classification of enzymes acting on ureidoglycolate in biological databases

Database^a^	Release	EC 3.5.3.19	EC 4.3.2.3
GenBank Refseq	196	AllA (NP_415038) UAH (NP_199173)	DAL3 (NP_012298), UGL^b^ (WP_006480316)
UniProtKB/Swiss-Prot	110	DAL3 (P32459)	UGL^b^ (B1K3Y3)
		AllA (P77731)	
		UAH (Q8VXY9)	
Protein Data Bank	101	AllA (1YQC)	
Kegg	15-Apr-13	DAL3 AllA	UGL^b^
Enzyme	26-Jun-13	DAL3 AllA	UGL^b^
Brenda	01-Jul-13	DAL3 AllA UAH	UGL^b^
MetaCyc	17.1	UAH	DAL3 AllA
Proposed classification		UAH	DAL3 AllA

^a^Assignment in various databases of experimental characterized protein with urea- or ammonia-releasing activity on ureidoglycolate. Accession numbers are reported in parenthesis, when applicable.

^b^Identification of UGL of *Burkholderia cenocepacia* as ureidoglycolate lyase is based on sequence comparison of Edman degradation fragments obtained from a fractionated extract with UGL activity and apparently homogeneous in SDS-PAGE (22). As pointed out in previous analyses (23), several lines of evidence argue against a physiological role of this gene in ureidoglycolate metabolism: (i) experimentally characterized homologs have an established role in the metabolism of 4-hydroxyphenylacetate (24); (ii) the gene and its homologs are never observed in purine degradation clusters; (iii) in *B. cenocepacia*, genes encoding *bona fide* ureidoglycolate lyase are present immediately downstream allantoicase, the gene involved in the formation of ureidoglycolate from allantoate.

The confusion existing in the attribution of enzymatic activities on ureidoglycolate is reflected well by the DAL3 record in the dedicated *S. cerevisiae* genome database. While the corresponding protein is correctly described as urea-lyase and assigned to EC 4.3.2.3, it is also associated to a gene ontology class corresponding to the EC 3.5.3.19 reaction.

### The original sin

In the seventies, researchers studying the utilization of purine derivatives as a nitrogen source in *S. cerevisiae* made the interesting observation that genes involved in the pathway were clustered in the so-called ‘degradation of allantoin locus’ (DAL) on chromosome IX (26). The physical association of genes of purine degradation in yeast is similar to what is found in bacteria, but at variance with other fungi (27, 28). After the sequencing of the yeast genome, DAL remains the largest and best-understood example of a metabolic gene cluster in *S. cerevisiae* (29). Anyway, this story explains why a gene encoding ureidoglycolate lyase (*DAL3*) has been found for the first time in yeast, about 30 years ago (30). In that occasion, the yeast gene was called ‘ureidoglycolate hydrolase’, a name used as a synonym of ‘ureidoglycolate lyase’ or ‘ureidoglycolatase’ (Table 2) and not to mean that the enzyme had an ammonia-releasing activity (an activity not yet discovered at that time). The name ureidoglycolate hydrolase was also used in the publication of the DAL3 sequence in 1991 (31).

**Table 2.** Recommended and acceptable nomenclature for EC 3.5.3.19 and EC 4.3.2.3

	EC 3.5.3.19	EC 4.3.2.3
Catalysed reaction	Ureidoglycolate + H_2_O = 2NH_3_ + CO_2_ + glyoxylate	Ureidoglycolate = urea + glyoxylate
Accepted name	Ureidoglycolate hydrolase^a^	Ureidoglycolate lyase
Systematic name	(*S*)-ureidoglycolate amidohydrolase (decarboxylating)	(*S*)-ureidoglycolate urea-lyase (glyoxylate-forming)
Other used names	Ureidoglycolate amidohydrolase^b^	Ureidoglycolatase; ureidoglycolase; ureidoglycolate hydrolase^a^

^a^The use of this ambiguous name in databases and literature is deprecated.

^b^Recommended as the accepted name of EC 3.5.3.19.

However, in the meantime, a hydrolytic activity releasing ammonia from ureidoglycolate was discovered in soybean extracts (10). When classifying the plant activity, besides using the EC 3.5.3 class (amidinohydrolase) instead of the more appropriate EC 3.5.1 (amidohydrolase), the Enzyme Commission made an unwise decision to officially name the enzyme ureidoglycolate hydrolase, a name previously used in the literature to indicate a different enzyme (26, 30, 31). That was in 1992, and the trap for database curators was set.

**Figure 3.**
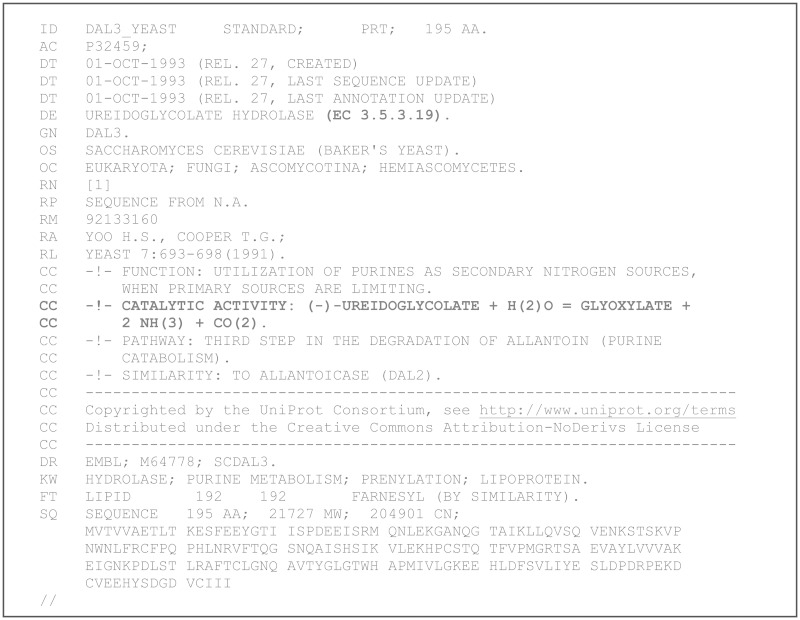
First documentary evidence of functional misassignment of DAL3/AllA proteins in database. The Swiss-Prot record shown was retrieved using the ‘History’ option of Uniprot (32). The text in bold represents interpolated information not present in the referred publication.

The first digital record in which the incorrect assignment of the enzyme activity is documented is the DAL3_YEAST entry of the release 27 (October 1993) of Swiss-Prot. In this record (Figure 3), the protein is named ureidoglycolate hydrolase according with the original publication, but with an interpolation of the evidence reported in the publication it is assigned to EC 3.5.3.19 and implicated in the catalysis of ammonia release from ureidoglycolate. Through the well-known process of automatic propagation of database annotations (32), this interpolation has been transferred to thousands of homologous genes. Unexpectedly, however, this interpolation has also been propagated in dedicated experimental studies of the protein.

### Incorporation of database interpolations in the scientific literature

After the determination of the DAL3 sequence and function, no further studies have addressed the activity of yeast protein. However, dedicated investigations have been carried out on homologous genes and proteins in other organisms, notably in *E. coli*. The genome of this bacterium revealed a locus of clustered genes for the utilization of allantoin. In the locus, expressed under anaerobiosis and nitrogen starvation, a gene (*allA*) was identified with similarity to *DAL3*; deletion of this gene abolished the activity on ureidoglycolate (34). Although in the metabolic scheme reported in the article the enzyme was supposed to release urea, AllA was named ureidoglycolate hydrolase according to the yeast protein and no explicit reference to the EC class was made. Incidentally, the assay used to monitor the activity, based on the observation of the glyoxylate product, did not allow one to distinguish between the two different activities. Therefore, an occasion for correcting the wrong information in the databases was missed and the AllA annotation provided further support for the assignment of these enzymes to EC 3.5.3.19.

Some years later, the AllA protein had its 3D structure determined in the frame of structural genomics, together with several other proteins of *E. coli* O157:H7. The published AllA structure revealed two gracious small barrel (cupin) folds. Along with a fully coloured illustration of the AllA protein, the authors wrote down the reaction catalysed by the enzyme as it was reported in all databases: ureidoglycolate and water gives two ammonia, one carbon dioxide and glyoxylate (8). Later on, in biological databases, this evidence was used as a basis for the assignment of DAL3/AllA to EC 3.5.3.19. The circle was completed.

### Error mechanisms in the digital monk era

The following section does not represent original research, but the adaptation of categories and explanations already established in the philological studies of handwritten manuscripts (1, 2).

#### Opacity of the model

A major cause of interpolations in the work of amanuenses was the presence of obscure parts in the original manuscript. In such cases, the amanuensis could have left a blank space in the manuscript or try to interpret the content of the illegible part, most often introducing an erroneous innovation, i.e. a corruption. In the article describing the gene and protein sequence of DAL3 (31), neither the catalysed reaction nor the EC number of the enzyme was explicitly stated. Therefore, this information had to be interpolated by the database curator based on previous literature and the enzyme name (see Figure 3).

#### Saut du même au même

The presence in the written text of similar or identical words at close distance is a frequent occasion of errors in manuscript transcriptions. The text following the second occurrence of the word could be attached to the first one and the part in the middle omitted when the reading of the scribe ‘jumped’ between two identical words (‘Saut du même au même’). Many different enzymes have similar parts in their name (they are, by the way, all ending in -ase), providing occasions for confusion and misclassification. In this particular case, the name used in the publications of the *DAL3* gene and sequence (30, 31)—ureidoglycolate hydrolase—was identical to the name officially assigned by the Enzyme Commission to another EC number (see Table 2).

#### Entropy or presupposition

When the transcription of a new manuscript is started, the entropy is at its higher level. With the proceeding of the transcription, the recognition of the narrative context lowers the entropy and also increases the possibility that the amanuensis makes assumptions and deviates from the model. Inspection of AllA_ECOLI records in Swiss-Prot (release 40, October 2000) reveals that the database curator followed the model (31) in reporting peculiar features of the *E. coli* protein (such as the induction by glyoxylate), but deviated from the model when assigning the protein to EC 3.5.3.19, probably due to the presupposition created by the annotation of the DAL3 protein in the database.

### Error persistence and good practice for correction

**Figure 4.**
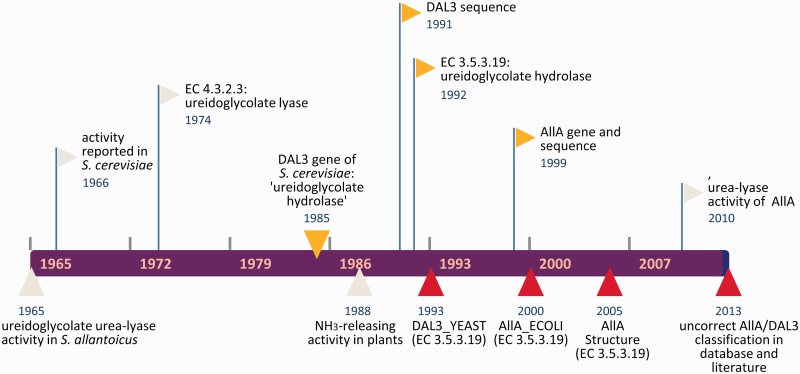
Timeline of DAL3/AllA misclassification as EC 3.5.3.19. Events marked in red represent erroneous interpolation in database or literature; events marked in yellow represent causes or occasions of errors.

The series of events that our analysis has identified as relevant for the misclassification of DAL3/AllA genes as EC 3.5.3.19 is summarized in Figure 4. What is surprising in this figure is the long persistence of the erroneous interpolation, which was initially introduced in the databases about 20 years (and 90 releases) ago. This is in spite of the fact that DAL3/AllA proteins have been correctly described as urea-lyase in several works (28, 29, 34, 35) and their activity directly demonstrated by experiments (14). However, it should be noted that no sequence information on DAL3/AllA protein with the correct assignment to EC 4.3.2.3 based on experimental evidence has been submitted to the database, a condition that has likely contributed to the error persistence. In other words, the literature evidence may not be sufficient for a rapid emendation of erroneous information firmly established in databases, but direct intervention on the databases is required.

Based on the above considerations in this and similar cases, the following actions should be considered to ensure rapid emendation of the database information. (i) Submission of data with a correct evidence-based annotation to the sequence databases. (ii) Direct communication to the database curators of the evidence for a correct functional classification. We have submitted a Third Party Annotation (TPA: experimental) of the *E. coli* AllA protein to GenBank according to the experimental results presented in Figure 2. We have used the feedback form of the ALLA_ECOLI record of Uniprot to report evidence for the protein assignment to EC 4.3.2.3. In addition, we plan to email curators of the other databases listed in Table 1 the result of the present analysis.

### Concluding remarks

The aim of our analysis was not to blame errors of database curators. On the contrary, we have pointed out the existence of confounding elements in enzyme classification and previous literature that explain or justify the unfortunate interpolation introduced in the databases. We know by personal experience that interpreting the conflicting data existing on the ureidoglycolate metabolism may pose problems even to specialists in purine catabolism. We have thus noted with appreciation that correct classification of genes and enzymes is found on this subject in an electronic source containing >2000 pathways (25). To disentangle such intricate and contrasting information beyond the evidence reported in biological databases certainly requires careful and critical reading of the literature sources.

One aim of this work was to ensure rapid emendation of the classification of these gene and enzymes in biological database and avoid a recurrence of the error in the scientific literature. The distinction of the two different mechanisms of nitrogen release by the enzymes acting on ureidoglycolate is not simply a matter of enzyme classification. More importantly, the awareness of the form of nitrogen released from ureidoglycolate, together with information on the presence and activity of urease, is relevant for the understanding of the metabolism purine-derived nitrogen in several organisms. This notion can also be relevant for the understanding of pathogenic mechanisms involving production of ammonia and alkalinization of the microbial environment. Known pathogens in which urea metabolism and ammonia production constitute virulence factors are the bacteria *Helicobacter pylori* and *Proteus mirabilis* (36–38), and the fungi *Cryptococcus neoformans* and *Coccidioides posadii* (39, 40). The ‘ureidoglycolate hydrolase’ (UGH) protein of *C. posadii*, a pathogen causing life-threatening respiratory disease in humans, has been recently reported as a possible virulence factor contributing to the production of ammonia at pulmonary sites (7). However, the phenotypes observed in Δ*UGH* and Δ*UGH*/Δ*URE* (urease) deletion mutants have been interpreted according to the incorrect assignment of this *DAL3* homolog to EC 3.5.3.19. These results should be reinterpreted in the light of the following evidence: (i) The *C. posadii* enzyme is expected to release urea, not ammonia, from ureidoglycolate; (ii) ureidoglycolate is moderately stable and decays spontaneously to glyoxylate with the release of urea; this latter observation may provide an explanation for the delayed kinetics of ammonia production in Δ*UGH* strains (7).

Another aim of our work was to point out the possible existence of common error mechanisms in the manual annotation of biological database. Similar patterns or mechanisms have been identified in the studies of the transmission of knowledge in the time before computers, the Internet and printed books. We are aware that, at variance with the amanuensis transcriptions of handwritten manuscripts, the manual transfer of previous information in a digital form always requires interpretation and analysis of the original source (which is often published evidence). This, however, means that there are more chances for the introduction of modifications of the model. Certainly, most of the time the interpolation introduced by the curator is correct, but there are occasions of erroneous interpolations that can affect subsequent database annotations and the scientific literature. In particular, our analysis has identified the opacity of the model (i.e. the lack of biochemical details in the original publication) as the cause for the introduction of an interpolation by the database curator, and the use of an identical name for two different activities as an occasion for the erroneous classification. By analysing the ENZYME database, we found 345 enzyme names used in more than one EC number. In the majority of cases, the shared EC numbers differ only for the last digit, meaning that the enzyme is essentially the same. However, in 106 instances, the EC numbers differ in the first three digits, and in 26 (including ureidoglycolate hydrolase) the difference pertains to the first digit, indicating a substantially different activity (Supplementary Table S1). As suggested by the example described here, such cases are expected to involve error and misclassification in databases and scientific literature.

The awareness of the causes of errors in manual annotation is important for database curators to identify issues that deserve particular attention in the examination of the original sources of information. This knowledge, however, could also be used for automatic control of the quality of data annotation. Quality control software is used in various databases to check at time of entry the consistency of the data, such as the correct translation of coding sequences or the presence of residues in allowed regions of the Ramachandran plot. With the recognition of common mechanisms of faulty annotation, the development of intelligent tools able to identify issues in gene and protein classification and limit the occurrence of ‘amanuensis’ errors can be envisaged.

## Methods

### Bioinformatics

Classification of DAL3, AllA and UGH has been visually inspected in the Genbank/GenPept (http://www.ncbi.nlm.nih.gov/genbank/), UniProtKB/Swiss-Prot (http://www.uniprot.org), PDB (http://www.rcsb.org), Kegg (http://www.genome.jp/kegg), Enzyme (http://enzyme.expasy.org/), Brenda (http://www.brenda-enzymes.org/) and MetaCyc (http://metacyc.org/) databases. The chronology of record submission to Genbank has been established by ordering ‘by date’ the list of entries ‘related to’ the genes or protein under analysis. The original DAL3_YEAST and ALLA_ECOLI records in Swiss-Prot have been retrieved using the ‘History’ option of the current Uniprot record. The search of enzyme names associated to different EC numbers has been carried out by parsing a local copy of the ENZYME database (retrieved from ftp.expasy.org/databases/enzyme) with the Perl script reported in Supplementary Table S1; the validity of the results has been checked by querying the official EC database (http://www.enzyme-database.org) with the enzyme names reported in the table.

### Biochemistry

The AllA protein was produced by recombinant expression in *E. coli* of the clone JW0493 of the ASKA collection (41). The histidine-tagged protein was purified by affinity chromatography (Talon resin) to apparent homogeneity as assessed by sodium dodecyl sulphate-polyacrylamide gel electrophoresis (SDS-PAGE) analysis. The unstable ureidoglycolate substrate was generated in a pre-reaction using allantoate and recombinant allantoate amidohydrolase (AAH) from *E. coli* and ureidoglycine aminohydrolase (UGLYAH) from *A. thaliana* (20). Allantoate was obtained from commercial allantoin (Sigma) through basic hydrolysis (42). The ammonia- or urea-releasing activity was monitored where monitored spectrophotometrically with a continuous assay coupled with glutamate dehydrogenase (EDH), in the presence or in the absence of jack bean urease (type c-3, Sigma). The typical reaction mixture comprised 1.6 µg/ml AAH and 6 µg/ml UGLYAH, 0.085 mM allantote, 0.1 mM MnCl_2_, 0.35 mM NADH, 2.5 mM α-ketoglutarate, 19.36 U of EDH (from bovine liver, Sigma) and 0.1 M potassium phosphate, pH 8. To assess metal dependency, the AllA protein (160 µg/ml) was incubated for 60 min with 0.1 mM EDTA (ethylenediaminetetraacetic acid). The metal in the pre-reaction mixture (containing Mn^2+^) was eliminated using a Chelex20 resin (1 mg for 1 ml of reaction mix) after the formation of ureidoglycolate. The activity was monitored of the EDTA-treated protein and of the EDTA-treated protein incubated for 30 min with an excess (0.3 mM) of various metal ions. The lack of urease inhibition was determined by adding urea at the end of the reaction.

## Supplementary Data

Supplementary Data are available at *Database* online.

Supplementary Data
