# Supplementary material for: Ureidoglycolate hydrolase, amidohydrolase, lyase: how errors in biological databases are incorporated in scientific papers and vice versa
Source: Database (Oxford). 2013 Oct 8;2013:bat071. doi: 10.1093/database/bat071 (PMC3793230; doi:10.1093/database/bat071)
Supplement: Supplementary Data [file supp_2013_bat071_index.html]

Ureidoglycolate hydrolase, amidohydrolase, lyase: how errors in biological databases are incorporated in scientific papers and vice versa — Supplementary Data 

# Ureidoglycolate hydrolase, amidohydrolase, lyase: how errors in biological databases are incorporated in scientific papers and vice versa

## Supplementary Data

files

**Files in this Data Supplement:**

- Supplementary Data - pdf file
