# Supplementary material for: Ureidoglycolate hydrolase, amidohydrolase, lyase: how errors in biological databases are incorporated in scientific papers and vice versa
Source: Database (Oxford). 2013 Oct 8;2013:bat071. doi: 10.1093/database/bat071 (PMC3793230; doi:10.1093/database/bat071)
Supplement: Supplementary Data [file supp_bat071_Table_S1.pdf]

Table S1. Enzyme names associated to more than one EC numbers differing in the first digit

**Enzyme name**[a]	**EC number**
5'-methylthioadenosine nucleosidase	2.4.2.28, 3.2.2.16
ACS	2.3.1.169, 4.2.3.42
Acetylornithinase	2.3.1.35, 3.5.1.16
Arginine decarboxylase	1.13.12.1, 4.1.1.19
CAD	1.1.1.195, 4.1.1.6
CYP17	1.14.99.9, 4.1.2.30
CYP17A1	1.14.99.9, 4.1.2.30
Chondroitinase	3.1.6.4, 3.1.6.12, 3.2.1.35, 4.2.2.5, 4.2.2.20, 4.2.2.21
Cobaltochelatase	4.99.1.3, 6.6.1.2
Gamma-glutamyltranspeptidase	2.3.2.2, 3.4.19.13
Glyceollin synthase	1.14.13.85, 2.5.1.36
Hyaluronidase	3.2.1.35, 3.2.1.36, 4.2.2.1
Hydroxyphenylpyruvate synthase	1.3.1.12, 5.4.99.5
Monophosphatidylinositol phosphodiesterase	3.1.4.11, 4.6.1.13
Nitrilase	3.5.5.1, 4.2.1.84
PAH	1.14.16.1, 3.5.3.22
PTE-2	2.3.1.176, 3.1.2.27
Phosphatidylinositol phospholipase C	3.1.4.11, 4.6.1.13
Propionyl-CoA carboxylase	4.1.1.41, 6.4.1.3
Pyruvate decarboxylase	1.2.4.1, 4.1.1.1
Spermidine dehydrogenase	1.5.99.6, 2.5.1.46
Squalene--hopene cyclase	4.2.1.129, 5.4.99.17
Thiaminase	2.5.1.2, 3.5.99.2
Trehalose synthase	2.4.1.245, 5.4.99.16
Tryptophanase	1.13.11.11, 4.1.99.1
Ureidoglycolate hydrolase	3.5.3.19, 4.3.2.3

[a] Names and EC numbers are from the ENZYME database data file (“enzyme.dat”) retrieved from ftp.expasy.org/databases/enzyme. The table was obtained by parsing in a Linux shell the enzyme.dat file with the following Perl command line scripts:

```
cat enzyme.dat | perl -ne 'next if(/Transferred entry|Deleted entry/); chomp; if (/^(DE|AN)\s+(.*)/) {($f,$n)=($1,$2); if($n=~/\.$/) {printf "$f   $o$n\n";$o=""} else {$o.=$n}} else {print "$_\n"}' | perl -ne 'chomp;$c=$1 if (/^ID\s+(.+)/); push(@{$n{$2}},$c) if (/^(DE|AN)\s+(.+)/); END {foreach $k (sort keys %n) { @A=@B=@{$n{$k}};if (scalar @A>1) {%h=map{$_ => s/(\d).\d+.\d+..+/$1/g} @B; printf "$k\t%s\n",join(", ",@A)  if (scalar keys %h>1)}}}'
```

The first pipe eliminates newlines in multi-line names and the second pipe does the parsing. Names sharing EC numbers differing in other positions than the first digit can be obtained by modifying the regular expression in the ‘map’ function. For instance, `map{$_ => s/(\d.\d+).\d+..+/$1/g}` gives the names sharing EC numbers differing at the first two digits.
